# Study of cellular heterogeneity and differential dynamics of autophagy in human embryonic kidney development by single-cell RNA sequencing

**DOI:** 10.1186/s12935-021-02154-w

**Published:** 2021-08-30

**Authors:** Chen Wen-jin, Pan Xiu-wu, Chu Jian, Xu Da, Chen Jia-xin, Chen Wei-jie, Wang Lin-hui, Cui Xin-gang

**Affiliations:** 1 Department of Urology, The Third Affiliated Hospital of Second Military Medical University, 700 North Moyu Road, Shanghai, 201805 China; 2 Department of Urology, Xinhua Hospital, Shanghai Jiaotong University, School of Medicine, 1665 Kongjiang Road, Shanghai, 200092 China; 3 Department of Urology, Gongli Hospital of Second Military Medical University, 219 Miaopu Road, Shanghai, 200135 China; 4 Department of Urology, Changzheng Hospital of Second Military Medical University, 415 Fengyang Road, Shanghai, 200003 China

**Keywords:** Human fetal kidney development, Autophagy, Single cell RNA sequencing, Heterogeneity, Wilms tumor

## Abstract

**Background:**

Autophagy is believed to participate in embryonic development, but whether the expression of autophagy-associated genes undergoes changes during the development of human embryonic kidneys remains unknown.

**Methods:**

In this work, we identified 36,151 human renal cells from embryonic kidneys of 9–18 gestational weeks in 16 major clusters by single-cell RNA sequencing (scRNA-seq), and detected 1350 autophagy-related genes in all fetal renal cells. The abundance of each cell cluster in Wilms tumor samples from scRNA-seq and GDC TARGET WT datasets was detected by CIBERSORTx. R package Monocle 3 was used to determine differentiation trajectories. Cyclone tool of R package scran was applied to calculate the cell cycle scores. R package SCENIC was used to investigate the transcriptional regulons. The FindMarkers tool from Seurat was used to calculate DEGs. GSVA was used to perform gene set enrichment analyses. CellphoneDB was utilized to analyze intercellular communication.

**Results:**

It was found that cells in the 13th gestational week showed the lowest transcriptional level in each cluster in all stages. Nephron progenitors could be divided into four subgroups with diverse levels of autophagy corresponding to different SIX2 expressions. SSBpod (podocyte precursors) could differentiate into four types of podocytes (Pod), and autophagy-related regulation was involved in this process. Pseudotime analysis showed that interstitial progenitor cells (IPCs) potentially possessed two primitive directions of differentiation to interstitial cells with different expressions of autophagy. It was found that NPCs, pretubular aggregates and interstitial cell clusters had high abundance in Wilms tumor as compared with para-tumor samples with active intercellular communication.

**Conclusions:**

All these findings suggest that autophagy may be involved in the development and cellular heterogeneity of early human fetal kidneys. In addition, part of Wilms tumor cancer cells possess the characteristics of some fetal renal cell clusters.

**Graphical abstract:**

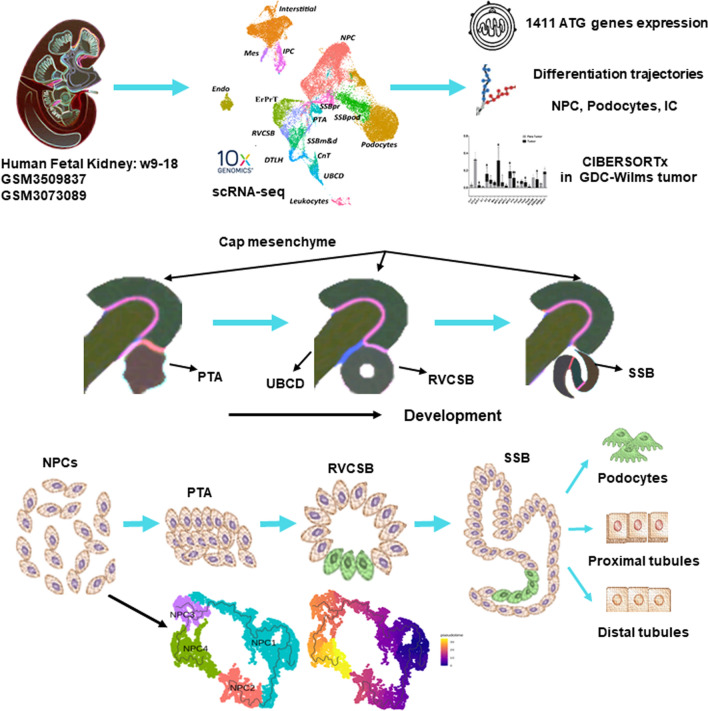

**Supplementary Information:**

The online version contains supplementary material available at 10.1186/s12935-021-02154-w.

## Introduction

The development of human kidneys starts in the intermediate mesoderm via communication between the ureteric bud (UB) and metanephric mesenchyme (MM), which are surrounded by mesenchymal cell clusters termed cap mesenchyme (CM) [1]. The mesenchyme-to-epithelium transition (MET) of CM is the essential for nephron morphogenesis [2]. Nephron progenitor cells (NPCs) originate from CM and transform into various nephrons through strict regulation of gene expression [3]. NPC niches present heterogeneity, where self-renewed NPCs are correlated with high-SIX2 expression but show slow cycling, while induced NPCs are correlated with low-SIX2 expression but show fast cycling [4]. Cells from NPCs gradually differentiate into several intermediate states: the pretubular aggregate (PTA), renal vesicle and comma-shaped body (RVCSB) and S-shaped body (SSB), which finally form the whole renal glomeruli and tubules. On the other hand, the collecting duct system derives from UB [5] and the interstitial progenitor cells (IPC) differentiate to form stromal cells, including interstitial cells (ICs), mesangial cells (Mes) [6]. MM also gives rise to the glomerular and vascular endothelial cells, into which eventually erythrocytes and leukocytes along with the bloodstream flow [7]. Previous study has revealed the underlying mechanisms of nephrogenesis [8], and both genetic and imaging techniques have identified almost all the cell types and their molecular characteristics during mouse kidney development [9, 10]. However, there exist species diversities in renal development or mature kidneys between rodents and humans [11]. Although several recent studies using single-cell RNA sequencing (scRNA-seq) to explore gene profiles have reported the molecular markers and dynamic gene expression in both rodents and humans [2, 5, 12–14], the cellular heterogeneity and corresponding gene pathways of initiating cells such as NPCs that are associated with the development of some renal diseases remain unclear.

Defects in nephrogenesis or other processes during fetal kidney development may predispose various renal abnormalities including Wilms tumor [15]. Wilms tumor is the most common pediatric renal cancer affecting 3–10 per million children and one in ten diagnosed children will die of this disease [1]. Wilms tumor appears to develop from nephrogenic abnormal structures, probably due to failure of conversion from mesenchyme to nephrons. Undifferentiated structures are rarely seen in normal kidneys but frequent noticed in Wilms’ tumor, resulting in cell unbounded growth and extensive tumorigenesis [16]. Many Wilms tumors are composed of mesenchyme and epithelial cells, which is consistent with the generally accepted hypothesis that these tumors originate from renal progenitor cells [15]. Earlier studies have genetically demonstrated that some Wilms tumor-associated mutations are also crucial in human kidney development, involving nephron or podocyte morphogenesis, such as WT1 [17–19]. However, no human fetal renal cell map has been reported to compare their features with those of Wilms tumor cells at the single-cell level. In addition, which type of kidney precursor gene signature is more likely to enrich in Wilms tumors remains unanswered.

Autophagy works via a lysosomal degradation process, which plays a vital role in orchestrating various human biological processes and homeostasis through transcriptional regulation of autophagy-related (ATG) genes [20]. These ATG genes have been reported to be evolutionarily conserved but functionally diversified [20, 21]. Autophagy is also highly active in cell differentiation, tissue or organismal development, whose functional significance has been previously demonstrated by validation of selective or systemic knock-out mammalian models of ATG genes during the past decades [22]. However, there is limited evidence about the role of autophagy in human fetal kidney development because the related studies have mainly focused on the relationship of autophagy with acute kidney injury, kidney fibrosis or tumors rather with the embryonic kidney [23, 24].

In this study, we used scRNA-seq analysis to globally investigate the dynamic expression of ATG genes among 23 heterogenetic cell types in human embryonic kidney development during the 9th to 18th gestational week. In addition, we used a combination of single-cell and bulk RNA-seq resolution to explore the correlation between human fetal renal cells and Wilms tumor samples, and found that the ATG gene profile of fetal renal cells was different from that of Wilms tumors. The findings of the present study may for the first time provide a novel perspective in exploring the link between human embryonic kidney development and autophagy. In addition, the relationship between the heterogenetic kidney precursor gene signature and Wilms tumor identified in this study suggests that some specific cell types in the embryonic stage may be the potential target for the treatment of Wilms tumors.

## Materials and methods

### Data acquisition

We performed multiple scRNA-seq analysis on human fetal kidneys. The scRNA-seq raw data were obtained from Semrau et al. [5] (GSM3509837; w9, w11, w13, w16, w18) and GSM3073089 (w17). To validate the scRNA analysis results in the Wilms tumor, two databases were included. Firstly, the scRNA-seq counts matrix of Wilms tumor was according to the report by Behjati et al. [25]. Secondly, the gene expression RNA-seq (FPKM) was acquired from GDC TARGET-WT, which consists of 126 Wilms tumor samples and 6 normal tissue samples, along with corresponding clinical information.

### scRNA-seq data processing and cell type identification

To control the quality of scRNA-seq raw data of the human fetal kidney samples, Seurat (version 3.2.2) was used, routinely excluding single cells with < 200 or > 6000 UMIs [26]. To remove batch effects among samples, Harmony (R package, version 1.0) was performed. The principal component dimension number was set as 50 with 2000 highly variable genes. Finally, 36,151 single human fetal renal cells were entered for downstream analysis.

For the sake of identifying major cell types, the FindAllMarkers code statement was used to label these single cells. Each cell cluster was recognized on the basis of the markers validated and published in previous studies using 10× Genomics scRNA-seq [2, 5, 14]. Markers in each major cluster are listed in Additional file 2: Table S1. The plot of the marker-labeled cell types is shown in Additional file 1: Figure S1.

### ATG genes collection

To explore the expression of ATG genes as fully as possible, a total of 1411 human ATG genes were obtained from the public database described by Tan et al. [27]. The inclusion criteria for ATG genes are as follows: (a) each ATG gene occurred in at least one of the Autophagy, Human Autophagy, Human Autophagy Modulators and THANATOS database; and (b) support from evidence of the literature on autophagy.

### Monocle3 for pseudotime analysis

In order to determine differentiation trajectories for major clusters with large cell number, such as NPCs, Pods and ICs, the R package Monocle 3 (version 0.2.3.0) designed by Cao et al. [28] was used, which has also been shown to be heterogeneous. The results were visualized as UMAP plot.

### Cell cycle analysis

Cell cycle activity plays an important role in development process. To calculate the cell cycle scores, involving G1, S and G2M, Cyclone tool of R package scran (version 1.18.1) was applied. The results were visualized into heatmap or barplot to show the proportion of cell cycle in different clusters or subgroups.

### Transcriptional factors analysis

We attempted to know the transcriptional regulons features during the development process. To investigate the transcriptional regulons (TFs) in the heterogenetic subgroups of particular types of cells. R package SCENIC [29] (version 1.2.2) was used. The adj. p value < 0.05 was considered as significant. The activity of top 10 regulons in each subgroup were displayed by heatmap.

### Analysis of differentially expressed genes (DEGs)

To explore the differentially expressed genes in each cluster during dynamic development process, the FindMarkers tool from Seurat was used to calculate DEGs. The adj. p value was set as < 0.05 and log_2_ [Fold change (FC)] was at least > 1. The results were visualized by volcano plot and applied to next analysis.

### Analysis of functional pathways enrichment

To find which functional pathways work in heterogenic cell clusters, we used GSVA (version 1.38.0) to perform gene set enrichment analyses based on C2, C5 and GO MSigDB gene sets [30, 31]. Pathways with adj. p value > 0.05 were not considered for further analysis. The upregulated and downregulated pathways were visualized by barplot or heatmap.

### Cell-to-cell communication

To investigate the communication among cell clusters by ligands and receptors, CellphoneDB was utilized to analyze intercellular communication based on Python algorithm [32]. We explored the cell-cell network among total 21 cell clusters (15 major clusters and their subgroups except leukocytes). The adj. p values < 0.05 of ligand–receptor pairs was considered as significant. We also used iTALK (R package, version 0.1.0) to visualize the network of major clusters as previously described [33].

### Abundance analysis in RNA-seq samples

To evaluate the correlations between the cell type in scRNA-seq Wilms tumor samples and bulk RNA-seq, CIBERSORTx (R version) was applied to detect the abundance of cell subgroups in GDC TARGET-WT expression matrix, which was under log2(TPM + 1) normalization. We used Student’s t test to detect differences in relative abundance of each cell type between the tumor and normal samples using GraphPad Prims 7.0 software.

### InferCNV

To investigate the in NPCs and NPCs-derived cell clusters or IPCs and ICs, InferCNV package (version 1.7.1) was used (Additional file 1: Fig. S2b, c). NPCs or IPCs were regarded as the control group and the cut-off value was set to 0.1.

### Statistical analysis

All statistical analyses and data visualization were conducted in R (version 4.0.1) and GraphPad Prism (version 7.0).

## Results

### Cell types identification and ATG genes expression overview

**Fig. 1:**
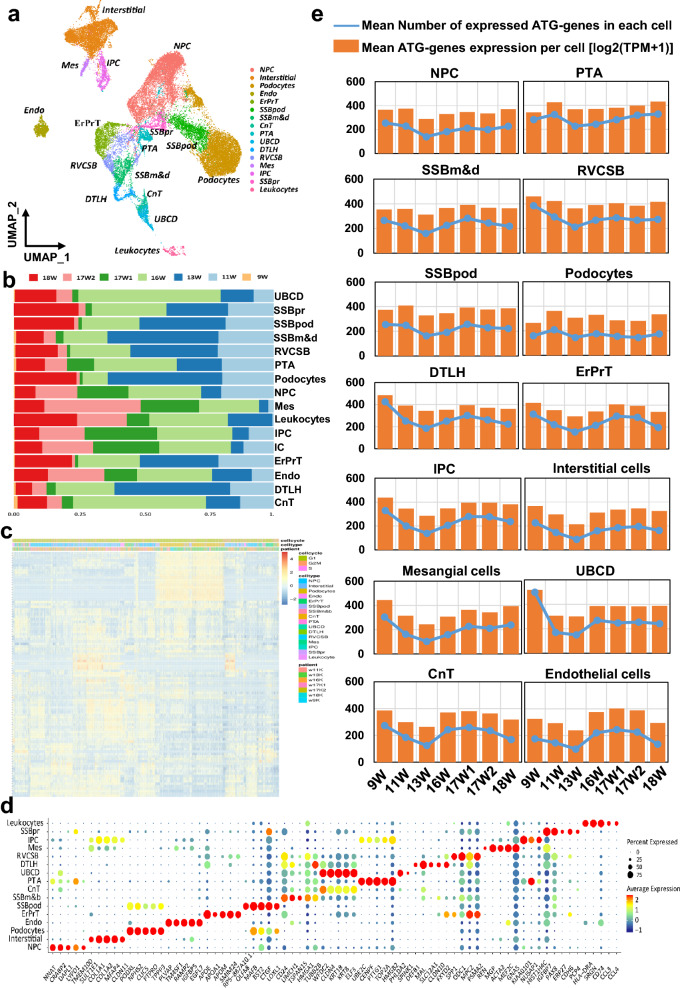
Cell types identification and dynamic expression of ATG genes in human fetal kidney. **a** UMPA plot of single cells mapped in this work differently colored by 16 main cell types. **b** Cell number of each main cell type fractioned by embryo weeks with different color annotations. **c** Overview for cell cycle scores (G1, G2M, S) of single cells clustered by cell types and embryo weeks with different color annotations. **d** Top 5 gene markers of 16 main cell types identified in this atlas. The bubble size presented the expressed percent, and the bubble color presented the average expression level of each gene. **e** Expressed ATG genes per single cell ranging from 9 to 18 weeks of four human fetal kidney. Blue line presents mean number of expressed ATG-genes in each cell; Orange bar presents mean ATG-genes expression per cell [log2(TPM + 1)]

After cell quality control, all cells were included in the analysis. Major cell types were identified by published markers [2, 5, 14]. Markers in each major cluster are listed in Additional file 2: Table S1. Of seven human fetal kidney samples including two 17-week samples, 36,151 single cells were retained after quality control (QC) and divided into 16 major cell clusters. In addition to endothelial cells and leukocytes, we identified 10 cap mesenchyme derived cells, including NPCs, PTA, RVCSB, SSB medial and distal cells (SSBmd), SSB proximal cells (SSBpr), SSB podocytes (SSBpod), podocytes (Pods), early proximal tubule (ErPrT), distal tubule and loop of Henle (DTLH), and connecting tubule (CnT); and three stromal derived cells, including IPCs, ICs and Mes, which were presented in the UMAP plot (Fig. 1a). The proportion of each cell type in different samples were visualized by barplot of Fig. 1b. Knowing that most fetal cells are under active renewal states, we calculated the cell cycle scores for each single cell, which were then clustered into main cell types (Fig. 1c). An overview of the cell cycle score heatmap showed no significant difference between different cell types, indicating that no cluster was affected by cell cycle. The top five marker genes of the major cell clusters were displayed in the bubble plot in Fig. 1d. To investigate ATG dynamic expression in different stages of human embryonic kidney development, we detected the expression level of ATG genes in each cell (Fig. 1e and Additional file 1: Fig. S2a). The expression data were scaled by Seurat and then normalized by log2(TPM + 1). The results showed that ATG genes were more likely to enrich in early weeks (w9), and they tended to decrease at w13 regardless of the cell type.

### Heterogeneous NPCs niche with different ATG levels uncovers the molecular mechanisms of initial nephron epithelium development

**Fig. 2:**
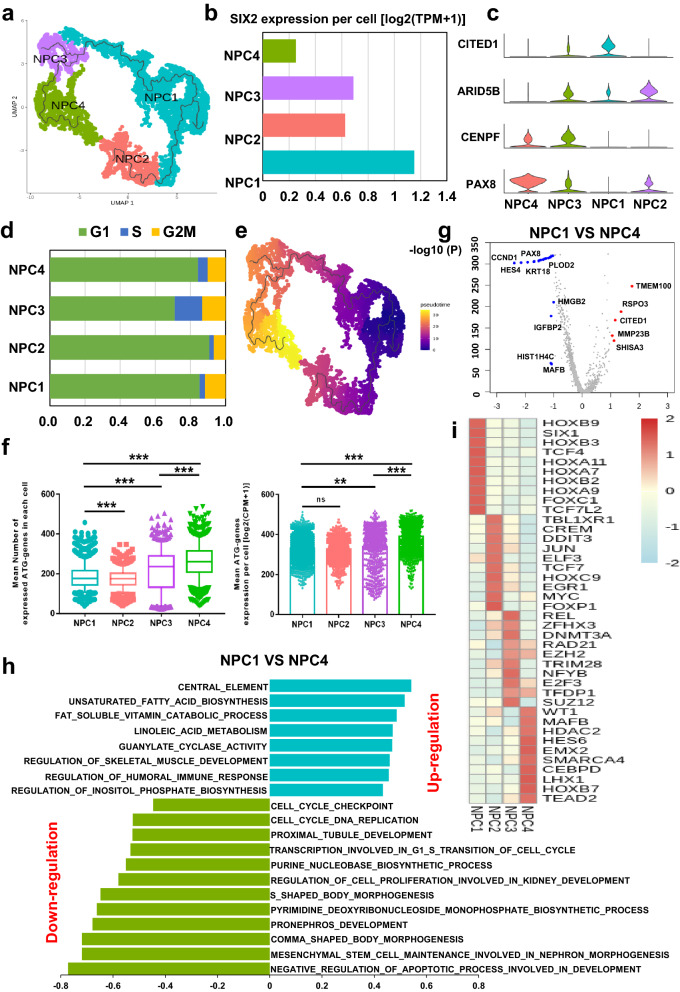
Heterogenetic NPCs niche present comprehensive gene expression pattern. **a** UMAP plot of NPCs subgroups of NPC1-NPC4 with different color annotations. **b** The SIX2 expression per cell [log2(TPM + 1)] in each single cell of four NPCs subgroups. **c** The violin plot presents marker genes of NPC1-NPC4, CITED1, ARID5B, CENPF, PAX8. **d** Cell cycle scores (G1, G2M, S) of each NPC subgroup. **e** The pseudotime trajectory plot of NPCs subgroups. The pseudotime showed the differentiation start to the end. **f** Different ATG expression of NPCs subgroups, ** represented P value < 0.01; *** represented P value < 0.001. **g** DEGs of NPC1 and NPC4: red dots represent upregulated genes in NPC1; blue dots represent down-regulated genes in NPC1. **h** GSVA results of NPC1 vs. NPC4. The left and right bar represented the downregulation and upregulation pathways respectively. **i** Top 10 enriched TFs in each subgroup of NPCs

Nephron epithelium formation initiates with the differentiation from NPCs to PTA and RVCSB. We used monocle 3 to perform pseudotime trajectory analysis on NPCs cluster. Based on the differentiation features, the NPCs were divided in to NPC1-4 four subgroups (Fig. 2a). Mammalian nephrogenesis is dependent on the regulation of transcriptional factors in homologous domains. Previous scRNA-seq study on fetal kidney development has genetically and Immunohistochemically demonstrated that SIX2 is a strong marker gene for NPC niche [13]. To investigate the heterogeneity of NPCs, we detected the SIX2 expression for NPC1-4 and found that NPC1 possessed the highest average level of SIX2, while SIX2 level of NPC4 was the opposite (Fig. 2b, Additional file 1: Fig. S3a). Then, we sought to find markers for the specific genes in each subgroup. The results showed that NPC1 expressed the most CITED1, while NPC4 had higher PAX8 expression but almost had no expression of CITED1 (Fig. 2c). CITED1 has also been reported to be strongly and positively expressed in cap mesenchyme cells [34], and relatively decrease from early NPCs to PTA or next step of nephrogenesis [5]. Additionally, PAX8 can be detected in late stages of cap mesenchyme, which could differentiate into CSB [35], and PAX8 + NPCs have morphogenesis potential [36]. Thus, we hypothesized that NPC1 (SIX2^high^CITED1^high^) and NPC4 (SIX2^low^CITED1^low^) could respectively derive from NPCs of earlier and later stage, while NPC2,3 could be the transient stage.

To validate which stage these clusters could be, we calculated the cell cycle scores for NPC1-4. As shown in Fig. 2d, NPC1 (SIX2^high^CITED1^high^) exhibited slower cycling and NPC4 (SIX2^low^CITED1^low^) showed faster cycling, which is consistent with the opinion about self-renewal (SIX2^high^, slow cycling) NPCs versus induced (SIX2^low^, fast cycling) NPCs [37]. In addition, the differentiation trajectory displayed that NPC1 and NPC4 could respectively represent the beginning and end of NPCs (Fig. 2e). The NPC4 potentially dominated in the induction of PTA, RVCSB. Next, we calculated the DEGs between NPC1 and NPC4 and found that RSPO3 and TMEM100 were highly regulated in NPC1 (Fig. 2g). RSPO3 is a typical stimulator of WNT pathway [38], and TMEM100 plays an important role in proliferation and apoptosis in fetal kidney development [39], suggesting that NPC1 tends to be self-renewing. The GSVA results also showed that the NPC1 pathway was enriched in cell cycle and nephron morphogenesis, while biosynthesis and induction pathways were enriched in NPC4 (Fig. 2h). Finally, the top 10 transcriptional factors analysis revealed that SIX1 and WT1 were respectively activated in NPC1 and NPC4 (Fig. 2i). SIX1 drives the differentiation of metanephric mesenchymal and cap mesenchymal cells but gradually decreases in the late stage [40]. The role of WT1 in fetal kidney tends to present induction effect, such as the regulation of MET and podocytes development and maturation [41]. When detecting the expression of ATG genes in NPC1-4, we found that the expression of ATG genes per cell in NPC4 was increased compared with NPC1 (P < 0.001, Fig. 2f).

### Podocytes are derived from SSBpod and present heterogeneity, whose subgroups possess diversified ATG genes expression

**Fig. 3:**
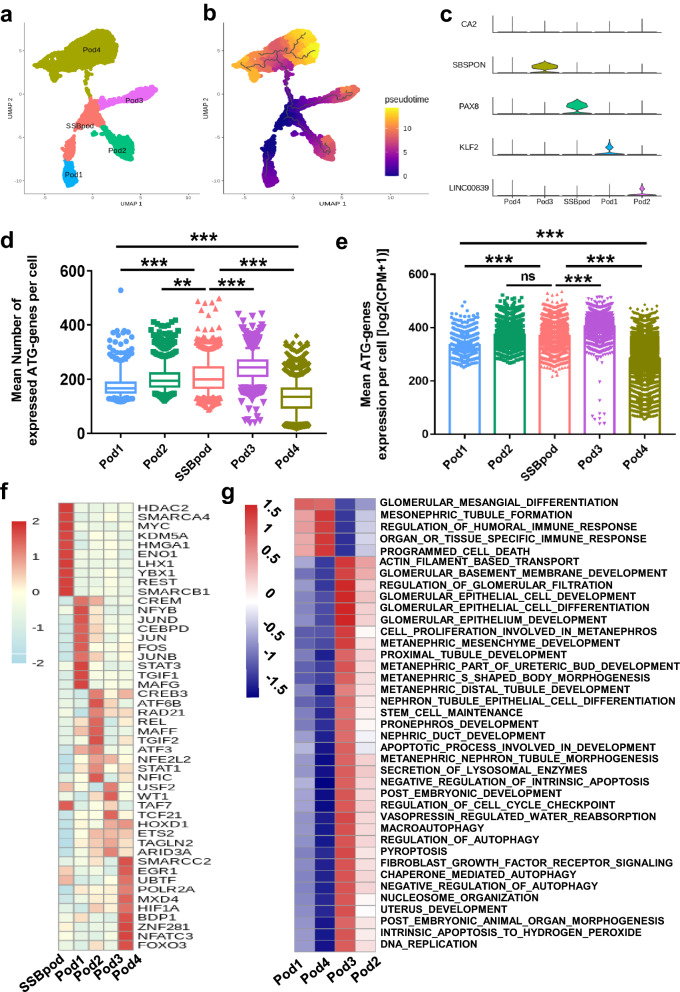
Heterogeneous podocytes present the complex gene expression pattern. **a** UMAP plot of SSBpod and podocytes subgroups, Pod1-Pod4. **b** The pseudotime trajectory plot of SSBpod and podocytes subgroups. The pseudotime showed the differentiation start to the end. **c** The violin plot presents marker genes of SSBpod and podocytes subgroups. **d**, **e** Different ATG expression of NPCs subgroups. ** represented P value < 0.01; *** represented P value < 0.001. **f** Top 10 enriched TFs in SSBpod and podocytes subgroups. **g** GSVA results of each podocytes subgroup. Red represent upregulated pathways; blue represent down-regulated pathways

Podocytes play a crucial role in renal filtration function, with the most cell number of all the clusters (8490) in our study. Previous literature reviewed the podocytes development and indicate that podocytes originated from the proximal differentiation branch of SSB [42]. Thus, our pseudotime analysis included the podocytes and SSBpod, which has been known as the intermediate state [5] or even precursors [43] of differentiation to podocytes. In Fig. 3a, the podocytes were classified into four subgroups based on monocle 3 marker genes. SSBpod was located in the beginning of this differentiation trajectory with Pod1-4 four directions to form podocytes. And Pod1 occurred in the end of trajectory with the highest cell number (Fig. 3b, Additional file 1: Fig. S3b). Then we detected the specific marker genes in SSBpod and each subgroup of podocytes (Fig. 3c). Compared with four podocytes subgroups, PAX8 became the distinct gene of SSBpod, which is a key factor involved in the process of kidney development via cell lineage determination and interaction [44]. KLF2 was the specific marker of Pod1 and participates in the physiological and pathological states in podocytes [45], suggesting that Pod1 could possess some general functions of podocytes. Then, we detected the expression of ATG gens in SSBpod and Pod1-4, and found that the expression of ATG genes per cell in Pod1, 2, 4 was decreased compared with SSBpod (P < 0.001, Fig. 3d, e). However, ATG expression level in Pod3 was higher than that in SSBpod. Finally, we investigated the transcriptional regulators (top 10) and enriched pathways to compare the potential functions between the subgroups. SCENIC analysis showed that HMGA1 was enriched in SSBpod (Fig. 3f), which is highly expressed in early embryogenesis but downregulated in differentiated in cells or tissues [46]. Immune-related TFs, such as NFYB, STAT3, MAFG, were enriched in Pod1 (Fig. 3f) and involved in inflammation caused by injury or death [47]. The GSVA results also revealed that immune-related or cell death pathways were enriched in Pod1 (Fig. 3g). Thus, Pod1 could develop into podocytes and participate in immune regulation [48]. The Pod3 possessed the highest ATG level, and renal-filtration-related pathways, autophagy pathways and kidney development associated pathways were enriched in this subgroup (Fig. 3g).

### IPCs differentiate into two heterogenetic ICs with different functions and ATG level

**Fig. 4:**
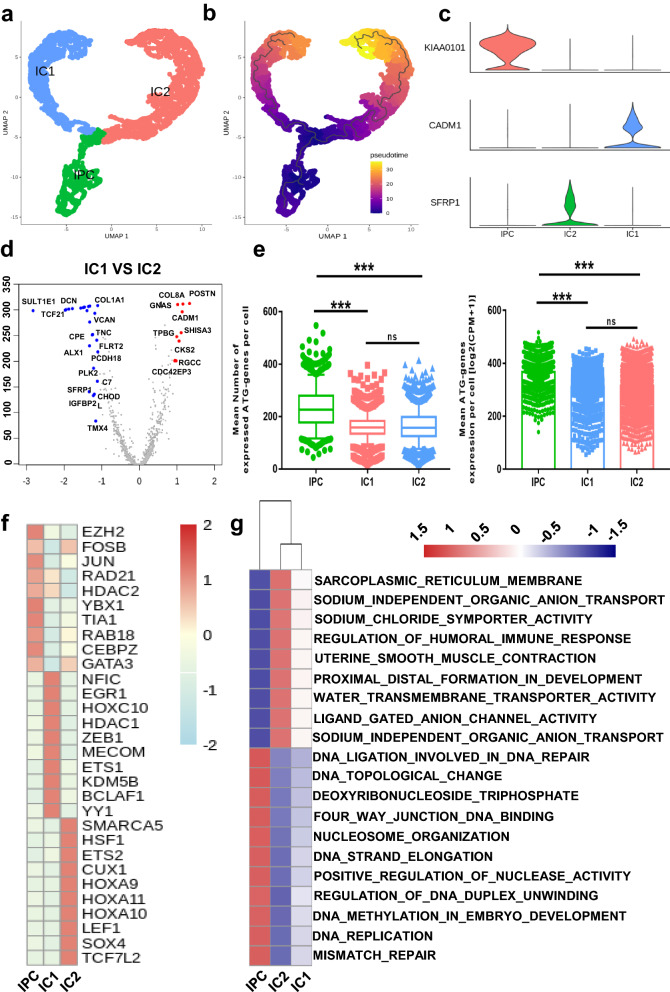
Heterogenetic interstitial cells present complex gene expression pattern. **a** UMAP plot of IPCs and ICs subgroups, IC1–IC2. **b** The pseudotime trajectory plot of IPCs and ICs subgroups. The pseudotime showed the differentiation start to the end. **c** The violin plot presents marker genes of IPCs and ICs subgroups. **d** DEGs of IC1 and IC2: red dots represent upregulated genes in IC1; blue dots represent down-regulated genes in IC1. **e** Different ATG expression of NPCs subgroups. ** represented P value < 0.01; *** represented P value < 0.001. **f** Top 10 enriched TFs in IPCs and ICs subgroups. **g** GSVA results of IPCs and ICs subgroups. Red represent upregulated pathways; blue represent down-regulated pathways

To evaluate the differentiation pattern of IPCs to ICs, we performed Monocle 3 for IPCs and ICs clusters as described in “Materials and methods” section. As shown in Fig. 4a, IPCs differentiated into IC1 and IC2 (Additional file 1: Fig. S3c), which was consistent with the pseudotime trajectory (Fig. 4b). The specific marker genes in the three clusters were KIAA0101, CADM1 and SFRP1 respectively (Fig. 4c). KIAA0101 is an important cell cycle regulated protein [49]. CADM1 as a cell adhesion molecule, can induce apoptosis of the renal epithelium [50]. SFRP1 plays an important role in renal interstitial parts formation [51]. To show the heterogeneity of IC1 and IC2 in DEGs, we visualized them in the volcano plot (Fig. 4d). Then, we detected the expression of ATG genes in IPC and IC1 & 2, and found that the expression of ATG genes per cell in ICs was decreased as compared with that in IPC, while there was no significant difference between IC1 and IC2 (Fig. 4e). Finally, we explored the transcriptional regulators (top 10) and enriched pathways to compare the potential functions among IPCs, IC1 and IC2. It was discovered that the dynamic regulation of EZH2 worked in the mouse model of renal mesenchyme formation, which was also enriched in IPCs (Fig. 4f). SOX4, which belongs to the SOXC family and is known to affect the fate of kidney development [52], was enriched in the IC2 (Fig. 4f). In addition, IPC presented pathways that symbolized the function of the progenitor such as activating DNA replication in embryo development. The function of ICs was enriched in IC2, such as regulating osmotic pressure through transmembrane transportation (Fig. 4g).

### Connection of cell clusters to WT RNA-seq and establishment of the NPC and IPC based regulatory network for human fetal renal cells

**Fig. 5:**
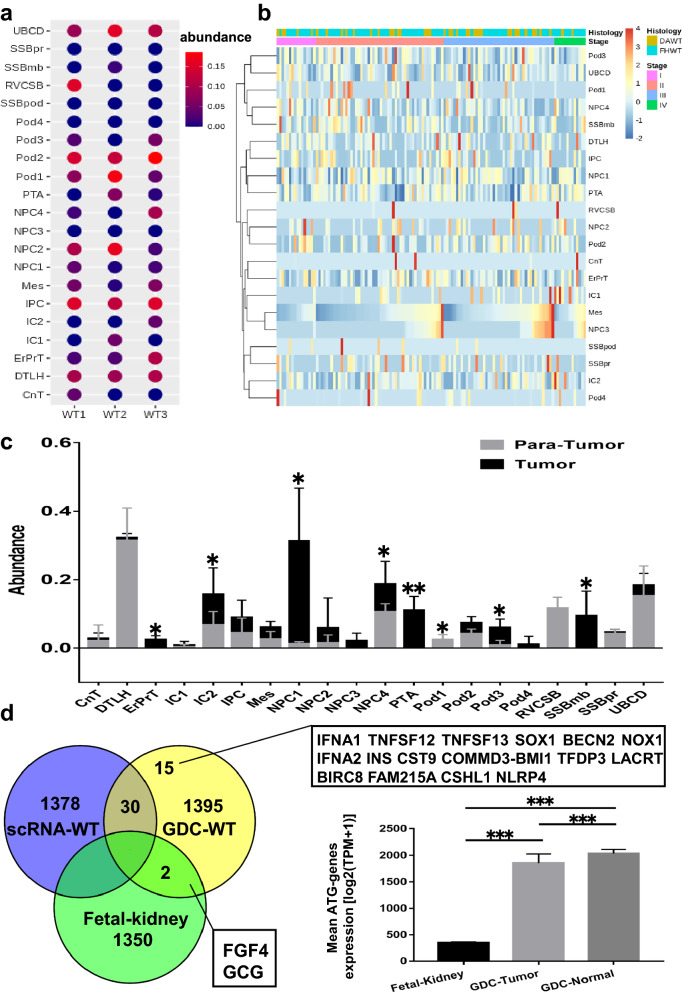
Characteristics of cell types identified by scRNA-seq in public WT RNA-seq cohort. **a** Relative abundance of cell types in WT scRNA-seq tumor samples calculated by CIBERSORTx. The bubble color presented the abundance of each cell type identified by scRNA-seq in the bulk RNA-seq samples. **b** Relative abundance of cell types in WT bulk RNA-seq tumor samples calculated by CIBERSORTx, clustered by histology and TNM stage. **c** Comparison of relative abundance between tumor samples and para-tumor samples from TARGET WT cohort. * represents p < 0.05; ** represents p < 0.01. **d** Venn diagram of ATG genes among three databases, 1395 of total 1411 ATG genes were recognized; overlapped genes were listed (left). The comparison of ATG expression among fetal kidney, TARGET tumor and normal samples. *** represents p < 0.001

The published literature has stated that renal stem cells in the embryo stage may develop to oncogenic stem cells leading to WT. To explore the potential tendency to developing WT of the cell clusters identified in this study, we assessed the abundance of each cell cluster in samples from WT scRNA-seq study [25] and GDC TARGET WT cohort by CIBERSORTx. As shown in Fig. 5a, WT1-3 were three samples from WT scRNA-seq study. The cell types in the present study displayed different fractions in each WT scRNA-seq sample. The abundance in GDC TARGET WT samples was normalized by Z-score in Fig. 5b. Each sample had distinct cell types factions, and the higher-abundance sample tended to have favorable histology WT (FHWT). Then, we detected abundance differences of different cell types between matched tumor and para-tumor samples from GDC TARGET WT. It was found that the fraction of NPC1, PTA, ErPrT and IC2 was significant higher in tumor samples than that in para-tumor samples (Fig. 5c), suggesting that the characteristics of these cell types tended to be similar to some tumor derived cells in WT. Next, we compared the ATG genes expression in fetal kidney, scRNA-WT and GDC-WT samples by Venn plot. It was found that some ATG gens were unique in GDC-WT tumors. In addition, the expression of ATG genes in tumor samples was significant different from that in normal samples or fetal kidney samples.

**Fig. 6:**
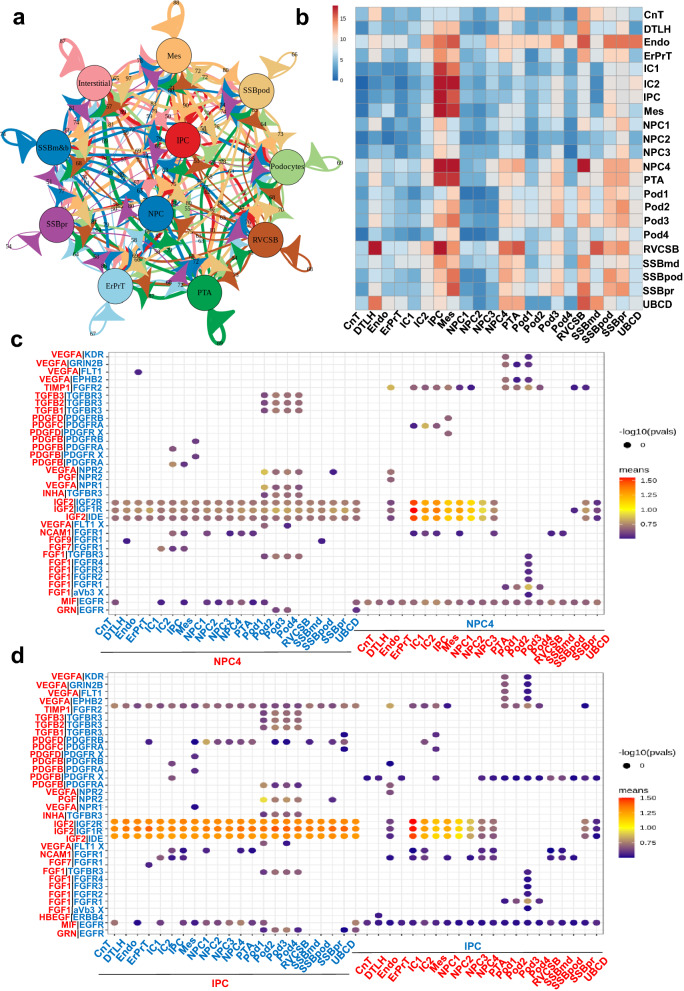
Cell–cell communication network in human fetal renal cell types. **a** The interaction network established by CellphoneDB; size and number of lines represents interaction counts. **b** Heatmap presents the number of potential ligand–receptor pairs between cell groups. **c** Bubble plots present ligand–receptor pairs of growth factors targeted to NPCs. The interactions intensity was presented by the bubble color. **d** Bubble plots present ligand–receptor pairs of growth factors targeted to IPCs. The interactions intensity was presented by the bubble color

To investigate the cell-cell communication network between the cell types of nephrogenesis in the study, we applied CellphoneDB (python version) and iTALK R package to the scRNA-sEq. Notably, NPCs and IPCs presented the most interactions with the other cell clusters (Fig. 6a). The interaction density among different cell types was shown in Fig. 6b. Considering the above GSVA results and the features of fetal tissue cells, we collected CellphoneDB results on interaction pairs of growth factors. NPC4 possessed the highest interaction density compared with NPC1-3, and therefore we chose NPC4 cluster as the objective of ligand–receptor pairs in Fig. 6c. It was discovered that IGF2 produced by NPC4 could interact with the other cell clusters via IGF2R, IGF2R and IDE, and NPC4 interacted with podocytes by TGFβ family. The receptors of IGF2 on NPC4 could strongly interact with ErPrT, ICs and IPCs. The profile of interactions pairs in IPC was relatively different from NPC, but the IGF2 related ligand–receptor pairs played an important role in the interaction network of IPC (Fig. 6d).

## Discussion

In this article, we described a more detailed fetal renal cell atlas based on published scRNA-seq studies. Firstly, we discovered the heterogeneity of NPCs, ICs and podocytes and their corresponding differentiation directions and functions. Secondly, we investigated the dynamic expression of ATG genes in each cell type at different gestational weeks, and found that the ATG expression pattern was significantly different between subgroups of NPCs, ICs and podocytes. Thirdly, we explored the potential connection between fetal renal cell types and WT samples, and found that some cell clusters had distinct features between tumor and para-tumor samples, such as NPC1, ErPrT and IC2. Finally, we for the first time constructed an intercellular interaction network among cell types identified by scRNA-sEq. The results of the present study may provide new insights into the ATG expression profile during human fetal kidney development based on scRNA-seq, and cell types with more potential to develop to WT.

Autophagy is necessary for maintaining homeostasis and may participate in embryo development [53]. However, the underlying role of autophagy in human fetal kidney development remains elusive. On one hand, autophagy is mainly a prominent catabolism in cells by degrading abnormal proteins and impaired organelles. On the other hand, cells experience profound reprogramming involving changes in organelles and extracellular matrix composition. Previous study reviewed that autophagy exerted its effects in cell morphogenesis via cell cycle adjustment, adhesion molecule expression and TGFβ pathways [54]. These roles of autophagy were also consistent with our work. We found that cell cycle scores in NPCs presented heterogeneity, which was corresponded to different ATG genes expression and differentiation directions in NPC subgroups (Fig. 2d, f, l). Then, we detected CADM1, a known cell adhesion molecule, and found that it was a specific marker for IC1 (Fig. 4c), suggesting that autophagy may participate in the process of IPC to IC1. Finally, we also found in the cell–cell network that the TGFβ related ligand–receptor pairs played an remarkable role in IPC interactions with other cell types, which may provide novel perspectives for the study of the potential mechanism and regulation pattern of autophagy in human fetal kidney development.

Tumors originate from the development of abnormal somatic cells or immature stem cells [55]. Although there is no consensus on origin of WT, one possibility is that WT may derive from the malignant transformation of kidney progenitors or stem cells [56]. Earlier genomic, genetic and epigenetic studies reported that kidney progenitor cells or stem cells during the embryo stage had subsets similar to WT in terms of molecular marker expression [57]. In this study, we used CIBERSORTx tool to explore the correlation between fetal kidney cell types and WT dataset samples, and found that some cell types of human fatal kidneys such as NPC and PTA were enriched in WT tumor samples as compared with para-tumor samples. These enriched cell types were not only involved in kidney progenitors, stem cells or other fetal renal cells in the early stage. Meanwhile, we performed inferCNV by using NPCs and IPCs as control and found that NPC- and IPC-derived cells presented CNV differences (Additional file 1: Fig. S2b, c). For the first time, we validated the origin possibility of WT based on scRNA-sEq. However, two recent studies indicated that the RNA-seq of the original stem cells detected cancer-related genes due to contamination of gene sequence in mouse and cancer mutations are truly rare [58, 59]. Therefore, more studies are required to validate the connection between fetal renal stem cells or progenitors and WT tumor cells and clarify the underlying mechanism.

A recent scRNA-seq study on human fetal kidneys reported that there was a correlation between fetal renal cell types and a series of some other renal diseases except WT [2].Interestingly, we also identified some marker genes or TFs that were related to particular renal diseases; for instance PAX8 was found to be highly expressed in NPC4 and SSBpod (Figs. 2c and 3c), which was associated with subsequent differentiation based on functional analysis. PAX8 was found to affect the branching shape of a normally developing kidney and be expressed in metastatic renal cell carcinoma (RCC) and polycystic kidney disease [60]. We also found that HIF1α and FOXO3 were enriched in Pod4 (Fig. 3f). FOXO3 could respond to hypoxic stress and block HIF1-mediated apoptosis by regulating CITED2 in cancer cells [61], and HIF-1 related hypoxia pathways were acknowledged as working in RCC. In addition to NPCs and Pods, SOX4 was enriched in IC2, which was upregulated in RCC and related to poor prognosis [62]. Nevertheless, the main limitation of our work was to include both fetal renal cortex (17w) and the whole fetal kidney (w9, w11, w13, w16, w18), with the original intention was to include embryonic kidney scRNA-seq data of all published representative weeks. This deserved discussion though the combined data might not affect the major conclusion (Fig. S4). In summary, the characteristics of fetal renal cells are potentially to be the targets to multiple renal diseases, though it needs to be validated in vitro and through clinical trials.

In conclusion, for the first time we uncovered the dynamic expression of ATG genes in the development of human fetal kidneys, and this finding may promote future study on the comprehensive mechanism of autophagy in human fetal kidney development. scRNA-seq revealed the novel heterogeneous fetal renal cells that possess different ATG levels and diverse function patterns. For the first time, we demonstrated that progenitor cells were potential during the process of conversion to WT during the fetal stage by using sing-cell and bulk RNA-sEq. In addition, we profiled a new map of fetal renal cells to explain heterogeneous biological functions by using scRNA-seq, which may provide new targets for the treatment of renal diseases.

## Supplementary Information

**Additional file 1: Figure S1.** The markers labeledcell types plot. **Figure S2. A** Expressed ATG genes per single cell in SSBprand leukocytes.** B**,** C** inferCNV results of NPC and IPC derived fetal renalcells. **Figure S3. A**SIX2 expression in Monocle 3 cluster of NPCs.** B** IL1R1 expression in Monocle 3cluster of SSBpod and pods. (IL1R1 is the marker of SSBpod).** C** CENPKexpression in Monocle 3 cluster of IPC and ICs. (CENPK is the marker of IPC). **Figure S4.** UMAP plot of different samples in all weeks.

**Additional file 2: TableS1.** Markers for eachmajor cluster are listed in this file.

## Data Availability

The scRNA-seq raw data of human fetal kidney has been online GSM3509837 (w9, w11, w13, w16, w18) and GSM3073089 (w17). The scRNA-seq counts matrix of Wilms tumor has been reported by Behjati et al. The gene expression RNA-seq (FPKM) was acquired from GDC TARGET-WT.

## Abbreviations

NPC: Nephron progenitor cells
PTA: Pretubular aggregate
RVCSB: Renal vesicle and comma-shaped body
SSBm&d: S-shaped body, medial and distal segments
SSBpr: S-shaped body, proximal segment
SSBpod: S-shaped body, proximal segment (podocyte precursor cells)
Pod: Podocytes
DTLH: Distal tubule and loop of Henle
ErPrT: Early proximal tubule
CnT: Connecting tubule
IPC: Interstitial progenitor cells
IC: Interstitial cells
Mes: Mesangial cells
Endo: Endothelial cells

## Code availability

The software application was detailed described in “Materials and methods".

## References

[1] Little MH, Combes AN, Takasato M (2016). Understanding kidney morphogenesis to guide renal tissue regeneration. Nat Rev Nephrol.

[2] Wang P, Chen Y, Yong J, Cui Y, Wang R, Wen L, Qiao J, Tang F (2018). Dissecting the global dynamic molecular profiles of human fetal kidney development by single-cell RNA sequencing. Cell Rep.

[3] Short KM, Smyth IM (2016). The contribution of branching morphogenesis to kidney development and disease. Nat Rev Nephrol.

[4] O’Brien LL (2019). Nephron progenitor cell commitment: striking the right balance. Semin Cell Dev Biol.

[5] Hochane M, van den Berg PR, Fan X, Bérenger-Currias N, Adegeest E, Bialecka M, Nieveen M, Menschaart M, Chuva de Sousa Lopes SM, Semrau S (2019). Single-cell transcriptomics reveals gene expression dynamics of human fetal kidney development. PLoS Biol.

[6] Kobayashi A, Mugford JW, Krautzberger AM, Naiman N, Liao J, McMahon AP (2014). Identification of a multipotent self-renewing stromal progenitor population during mammalian kidney organogenesis. Stem Cell Rep.

[7] Abrahamson DR, Robert B, Hyink DP, St John PL, Daniel TO (1998). Origins and formation of microvasculature in the developing kidney. Kidney Int Suppl.

[8] Georgas K, Rumballe B, Valerius MT, Chiu HS, Thiagarajan RD, Lesieur E, Aronow BJ, Brunskill EW, Combes AN, Tang D (2009). Analysis of early nephron patterning reveals a role for distal RV proliferation in fusion to the ureteric tip via a cap mesenchyme-derived connecting segment. Dev Biol.

[9] Brunskill EW, Park J-S, Chung E, Chen F, Magella B, Potter SS (2014). Single cell dissection of early kidney development: multilineage priming. Development.

[10] Mugford JW, Yu J, Kobayashi A, McMahon AP (2009). High-resolution gene expression analysis of the developing mouse kidney defines novel cellular compartments within the nephron progenitor population. Dev Biol.

[11] Little MH (2015). Improving our resolution of kidney morphogenesis across time and space. Curr Opin Genet Dev.

[12] Lindström NO, Guo J, Kim AD, Tran T, Guo Q, De Sena Brandine G, Ransick A, Parvez RK, Thornton ME, Baskin L (2018). Conserved and divergent features of mesenchymal progenitor cell types within the cortical nephrogenic niche of the human and mouse kidney. J Am Soc Nephrol.

[13] Lindström NO, Tran T, Guo J, Rutledge E, Parvez RK, Thornton ME, Grubbs B, McMahon JA, McMahon AP (2018). Conserved and divergent molecular and anatomic features of human and mouse nephron patterning. J Am Soc Nephrol.

[14] Combes AN, Zappia L, Er PX, Oshlack A, Little MH (2019). Single-cell analysis reveals congruence between kidney organoids and human fetal kidney. Genome Med.

[15] Schedl A (2007). Renal abnormalities and their developmental origin. Nat Rev Genet.

[16] Rivera MN, Haber DA (2005). Wilms’ tumour: connecting tumorigenesis and organ development in the kidney. Nat Rev Cancer.

[17] Gessler M, Poustka A, Cavenee W, Neve RL, Orkin SH, Bruns GA (1990). Homozygous deletion in Wilms tumours of a zinc-finger gene identified by chromosome jumping. Nature.

[18] Haber DA, Buckler AJ, Glaser T, Call KM, Pelletier J, Sohn RL, Douglass EC, Housman DE (1990). An internal deletion within an 11p13 zinc finger gene contributes to the development of Wilms’ tumor. Cell.

[19] Davies JA, Ladomery M, Hohenstein P, Michael L, Shafe A, Spraggon L, Hastie N (2004). Development of an siRNA-based method for repressing specific genes in renal organ culture and its use to show that the Wt1 tumour suppressor is required for nephron differentiation. Hum Mol Genet.

[20] Levine B, Kroemer G (2019). Biological functions of autophagy genes: a disease perspective. Cell.

[21] Klionsky DJ, Cregg JM, Dunn WA, Emr SD, Sakai Y, Sandoval IV, Sibirny A, Subramani S, Thumm M, Veenhuis M (2003). A unified nomenclature for yeast autophagy-related genes. Dev Cell.

[22] Mizushima N, Levine B (2010). Autophagy in mammalian development and differentiation. Nat Cell Biol.

[23] Fougeray S, Pallet N (2015). Mechanisms and biological functions of autophagy in diseased and ageing kidneys. Nat Rev Nephrol.

[24] Rybstein MD, Bravo-San Pedro JM, Kroemer G, Galluzzi L (2018). The autophagic network and cancer. Nat Cell Biol.

[25] Young MD, Mitchell TJ, Vieira Braga FA, Tran MGB, Stewart BJ, Ferdinand JR, Collord G, Botting RA, Popescu D-M, Loudon KW (2018). Single-cell transcriptomes from human kidneys reveal the cellular identity of renal tumors. Science.

[26] Butler A, Hoffman P, Smibert P, Papalexi E, Satija R (2018). Integrating single-cell transcriptomic data across different conditions, technologies, and species. Nat Biotechnol.

[27] Tan P, Ren Y, Zhang Y, Lin Y, Cui T, Huang Y, Zhang Y, Ning L, Yu J, Gao S (2019). Dissecting dynamic expression of autophagy-related genes during human fetal digestive tract development via single-cell RNA sequencing. Autophagy.

[28] Qiu X, Hill A, Packer J, Lin D, Ma Y-A, Trapnell C (2017). Single-cell mRNA quantification and differential analysis with census. Nat Methods.

[29] Aibar S, González-Blas CB, Moerman T, Huynh-Thu VA, Imrichova H, Hulselmans G, Rambow F, Marine J-C, Geurts P, Aerts J (2017). SCENIC: single-cell regulatory network inference and clustering. Nat Methods.

[30] Subramanian A, Tamayo P, Mootha VK, Mukherjee S, Ebert BL, Gillette MA, Paulovich A, Pomeroy SL, Golub TR, Lander ES (2005). Gene set enrichment analysis: a knowledge-based approach for interpreting genome-wide expression profiles. Proc Natl Acad Sci.

[31] Liberzon A, Subramanian A, Pinchback R, Thorvaldsdóttir H, Tamayo P, Mesirov JP (2011). Molecular signatures database (MSigDB) 3.0. Bioinformatics.

[32] Vento-Tormo R, Efremova M, Botting RA, Turco MY, Vento-Tormo M, Meyer KB, Park J-E, Stephenson E, Polański K, Goncalves A (2018). Single-cell reconstruction of the early maternal-fetal interface in humans. Nature.

[33] Wang Y, Wang R, Zhang S, Song S, Jiang C, Han G, Wang M, Ajani J, Futreal A, Wang L (2019). iTALK: an R package to characterize and illustrate intercellular communication. BioRxiv.

[34] Boyle S, Misfeldt A, Chandler KJ, Deal KK, Southard-Smith EM, Mortlock DP, Baldwin HS, de Caestecker M (2008). Fate mapping using Cited1-CreERT2 mice demonstrates that the cap mesenchyme contains self-renewing progenitor cells and gives rise exclusively to nephronic epithelia. Dev Biol.

[35] Poleev A, Fickenscher H, Mundlos S, Winterpacht A, Zabel B, Fidler A, Gruss P, Plachov D (1992). PAX8, a human paired box gene: isolation and expression in developing thyroid, kidney and Wilms’ tumors. Development.

[36] Morizane R, Lam AQ, Freedman BS, Kishi S, Valerius MT, Bonventre JV (2015). Nephron organoids derived from human pluripotent stem cells model kidney development and injury. Nat Biotechnol.

[37] Short KM, Combes AN, Lefevre J, Ju AL, Georgas KM, Lamberton T, Cairncross O, Rumballe BA, McMahon AP, Hamilton NA (2014). Global quantification of tissue dynamics in the developing mouse kidney. Dev Cell.

[38] Pedraza C, Matsubara S, Muramatsu T (1995). A retinoic acid-responsive element in human midkine gene. J Biochem.

[39] Ren D, Ju P, Liu J, Ni D, Gu Y, Long Y, Zhou Q, Xie Y (2018). BMP7 plays a critical role in TMEM100-inhibited cell proliferation and apoptosis in mouse metanephric mesenchymal cells in vitro. In Vitro Cell Dev Biol Anim.

[40] Nie X, Xu J, El-Hashash A, Xu P-X (2011). Six1 regulates Grem1 expression in the metanephric mesenchyme to initiate branching morphogenesis. Dev Biol.

[41] Fanni D, Fanos V, Monga G, Gerosa C, Locci A, Nemolato S, Van Eyken P, Faa G (2011). Expression of WT1 during normal human kidney development. J Matern Fetal Neona.

[42] Yoshimura Y, Nishinakamura R (2019). Podocyte development, disease, and stem cell research. Kidney Int.

[43] Menon R, Otto EA, Kokoruda A, Zhou J, Zhang Z, Yoon E, Chen Y-C, Troyanskaya O, Spence JR, Kretzler M (2018). Single-cell analysis of progenitor cell dynamics and lineage specification in the human fetal kidney. Development.

[44] Sharma R, Sanchez-Ferras O, Bouchard M (2015). Pax genes in renal development, disease and regeneration. Semin Cell Dev Biol.

[45] Rane MJ, Zhao Y, Cai L (2019). Krϋppel-like factors (KLFs) in renal physiology and disease. EBioMedicine.

[46] Vignali R, Marracci S (2020). HMGA genes and proteins in development and evolution. Int J Mol Sci.

[47] Kuraishy A, Karin M, Grivennikov SI (2011). Tumor promotion via injury- and death-induced inflammation. Immunity.

[48] Bhargava R, Tsokos GC (2019). The immune podocyte. Curr Opin Rheumatol.

[49] Emanuele MJ, Ciccia A, Elia AEH, Elledge SJ (2011). Proliferating cell nuclear antigen (PCNA)-associated KIAA0101/PAF15 protein is a cell cycle-regulated anaphase-promoting complex/cyclosome substrate. Proc Natl Acad Sci USA.

[50] Kato T, Hagiyama M, Takashima Y, Yoneshige A, Ito A (2018). Cell adhesion molecule-1 shedding induces apoptosis of renal epithelial cells and exacerbates human nephropathies. Am J Physiol Renal Physiol.

[51] Matsuyama M, Nomori A, Nakakuni K, Shimono A, Fukushima M (2014). Secreted frizzled-related protein 1 (Sfrp1) regulates the progression of renal fibrosis in a mouse model of obstructive nephropathy. J Biol Chem.

[52] Huang J, Arsenault M, Kann M, Lopez-Mendez C, Saleh M, Wadowska D, Taglienti M, Ho J, Miao Y, Sims D (2013). The transcription factor Sry-related HMG box-4 (SOX4) is required for normal renal development in vivo. Dev Dyn.

[53] Wang L, Ye X, Zhao T (2019). The physiological roles of autophagy in the mammalian life cycle. Biol Rev Camb Philos Soc.

[54] Offei EB, Yang X, Brand-Saberi B (2018). The role of autophagy in morphogenesis and stem cell maintenance. Histochem Cell Biol.

[55] Liu J (2020). The “life code”: a theory that unifies the human life cycle and the origin of human tumors. Semin Cancer Biol.

[56] Brown KW, Malik KT (2001). The molecular biology of Wilms tumour. Expert Rev Mol Med.

[57] Pode-Shakked N, Dekel B (2011). Wilms tumor—a renal stem cell malignancy?. Pediatr Nephrol.

[58] Stirparo GG, Smith A, Guo G (2020). Cancer-related mutations are not enriched in naive human pluripotent stem cells. Cell Stem Cell.

[59] Avior Y, Lezmi E, Eggan K, Benvenisty N (2020). Cancer-related mutations identified in primed human pluripotent stem cells. Cell Stem Cell.

[60] Grimley E, Dressler GR (2018). Are Pax proteins potential therapeutic targets in kidney disease and cancer?. Kidney Int.

[61] Bakker WJ, Harris IS, Mak TW (2007). FOXO3a is activated in response to hypoxic stress and inhibits HIF1-induced apoptosis via regulation of CITED2. Mol Cell.

[62] Grimm D, Bauer J, Wise P, Krüger M, Simonsen U, Wehland M, Infanger M, Corydon TJ (2020). The role of SOX family members in solid tumours and metastasis. Semin Cancer Biol.

