# Supplementary material for: Study of cellular heterogeneity and differential dynamics of autophagy in human embryonic kidney development by single-cell RNA sequencing
Source: Cancer Cell Int. 2021 Aug 30;21:460. doi: 10.1186/s12935-021-02154-w (PMC8404318; doi:10.1186/s12935-021-02154-w)
Supplement: Supplementary file 1 — Additional file 1: Figure S1. The markers labeledcell types plot. Figure S2. A Expressed ATG genes per single cell in SSBprand leukocytes. B, C inferCNV results of NPC and IPC derived fetal renalcells. Figure S3. ASIX2 expression in Monocle 3 cluster of NPCs. B IL1R1 expression in Monocle 3cluster of SSBpod and pods. (IL1R1 is the marker of SSBpod). C CENPKexpression in Monocle 3 cluster of IPC and ICs. (CENPK is the marker of IPC). Figure S4. UMAP plot of different samples in all weeks. [file 12935_2021_2154_MOESM1_ESM.pdf]

Nephron progenitors

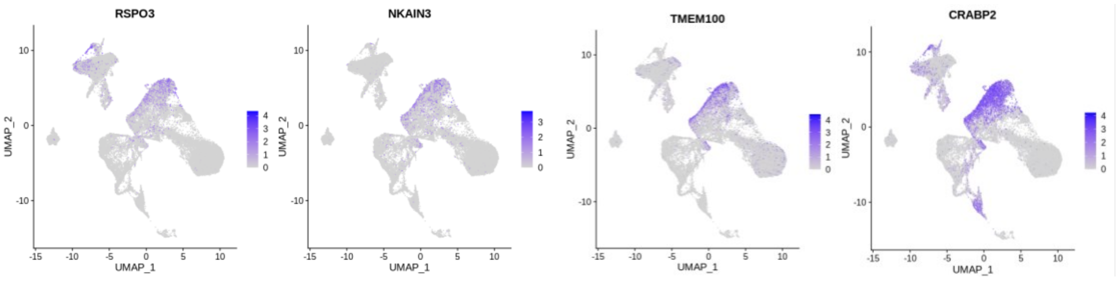

Pretubular aggregate

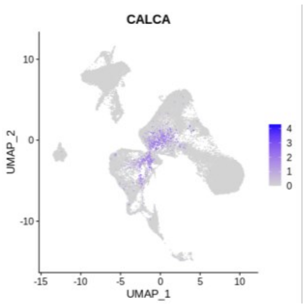

RVCSB

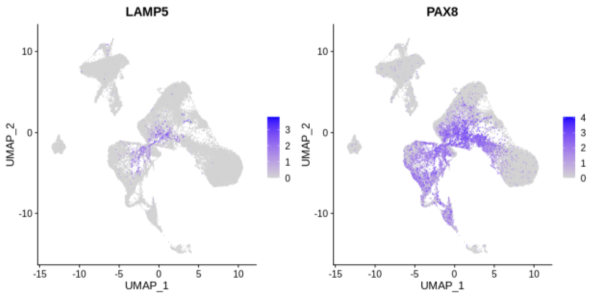

SSBpr

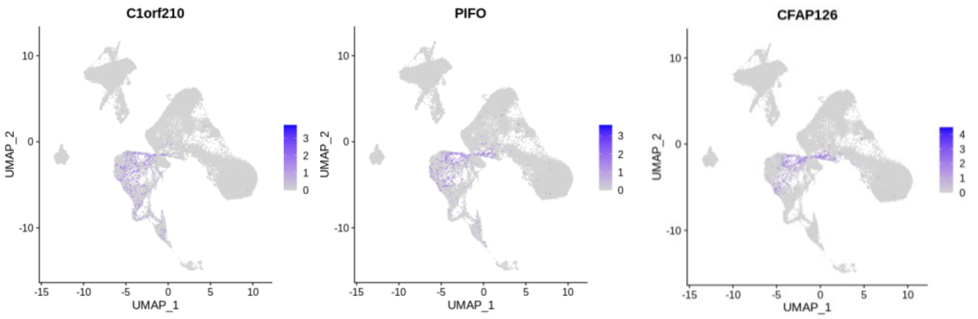

SSBpod

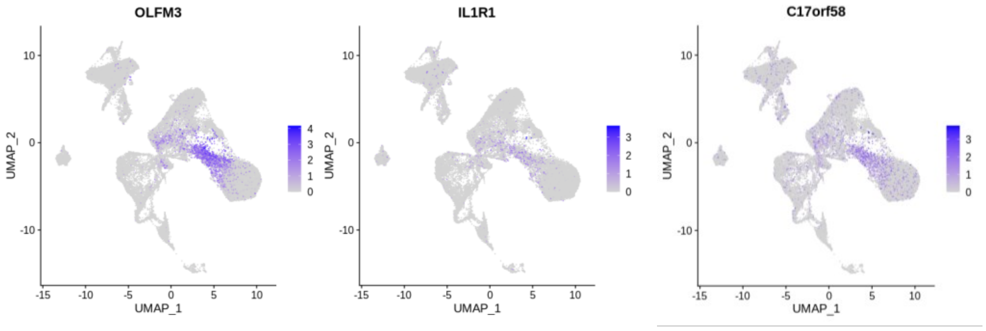

SSBm&d

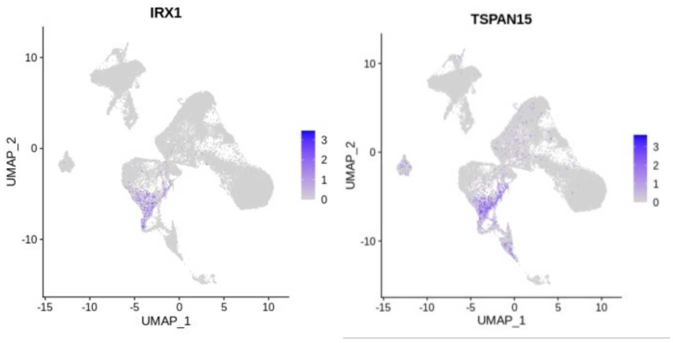

Podocytes

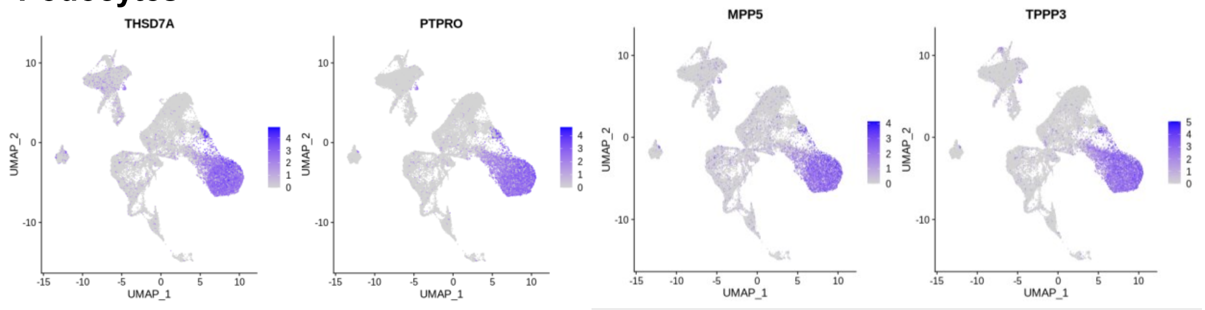

DTLH

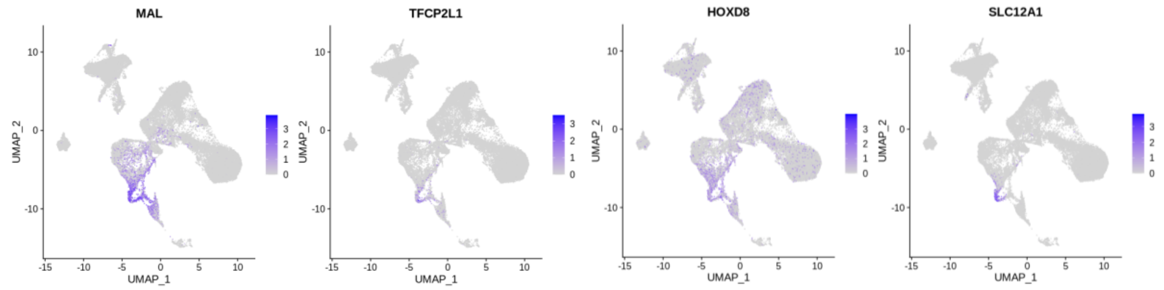

ErPrT

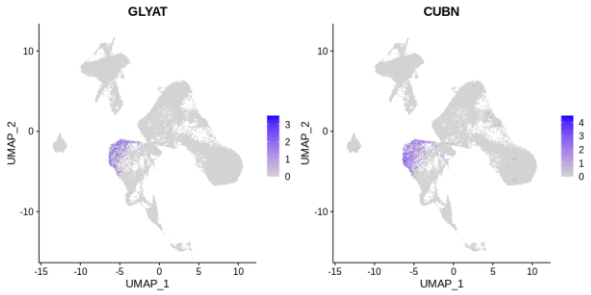

CnT

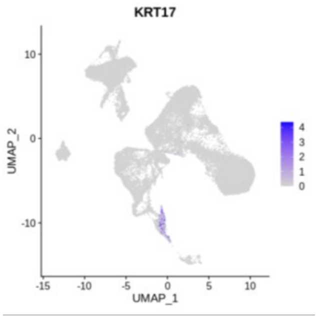

IPC

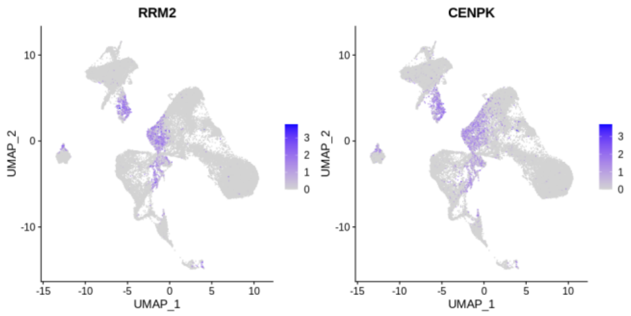

IC

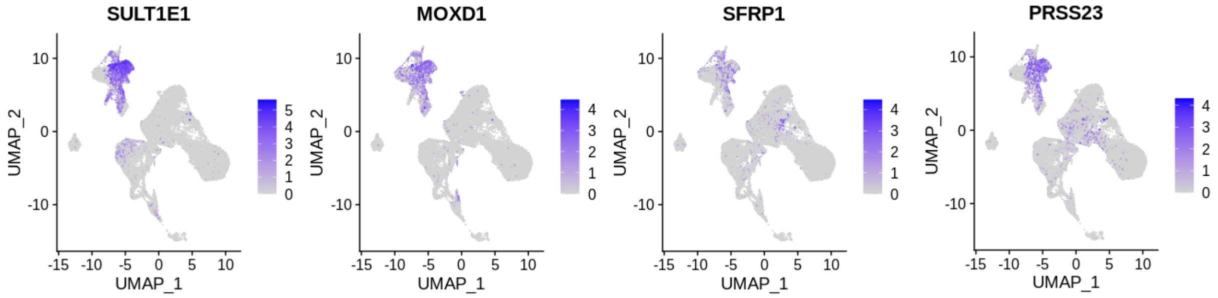

Mes

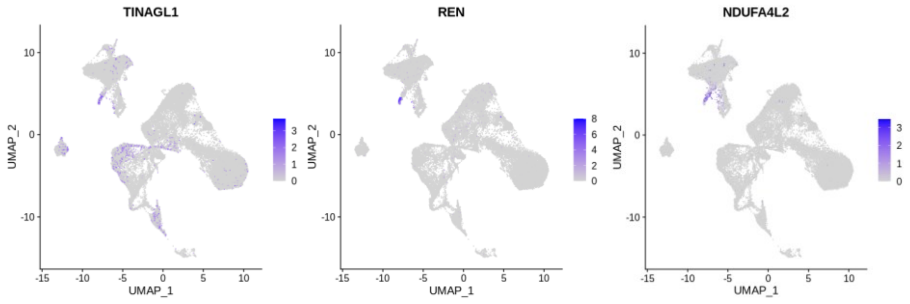

UBCD

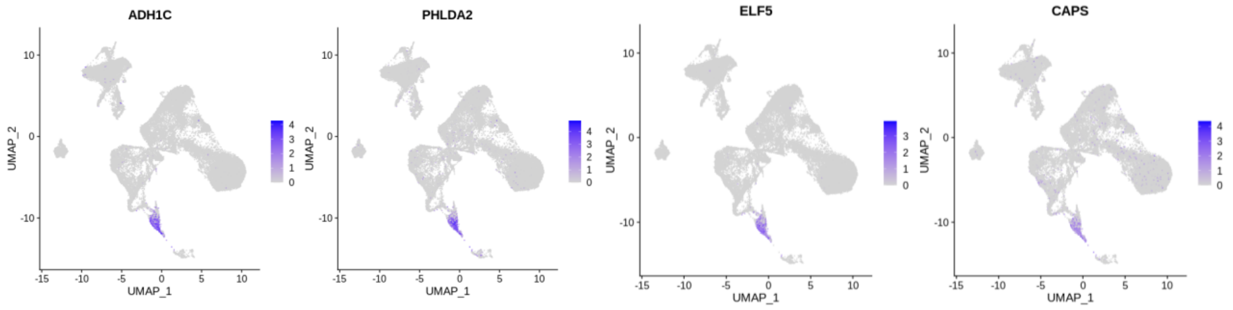

Endothelial cells

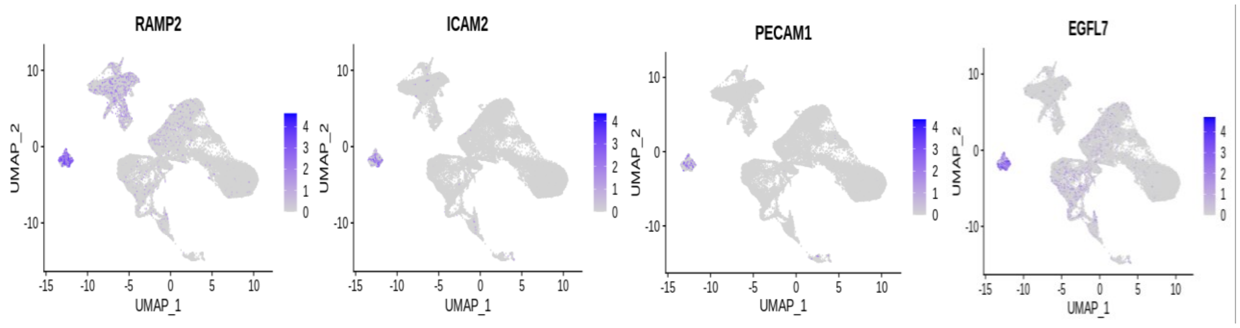

Leukocytes

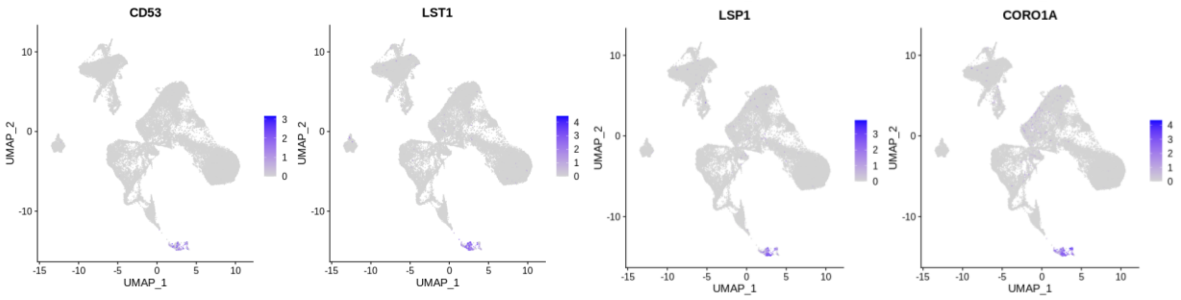

Fig.S1 The markers labeled cell types plot.

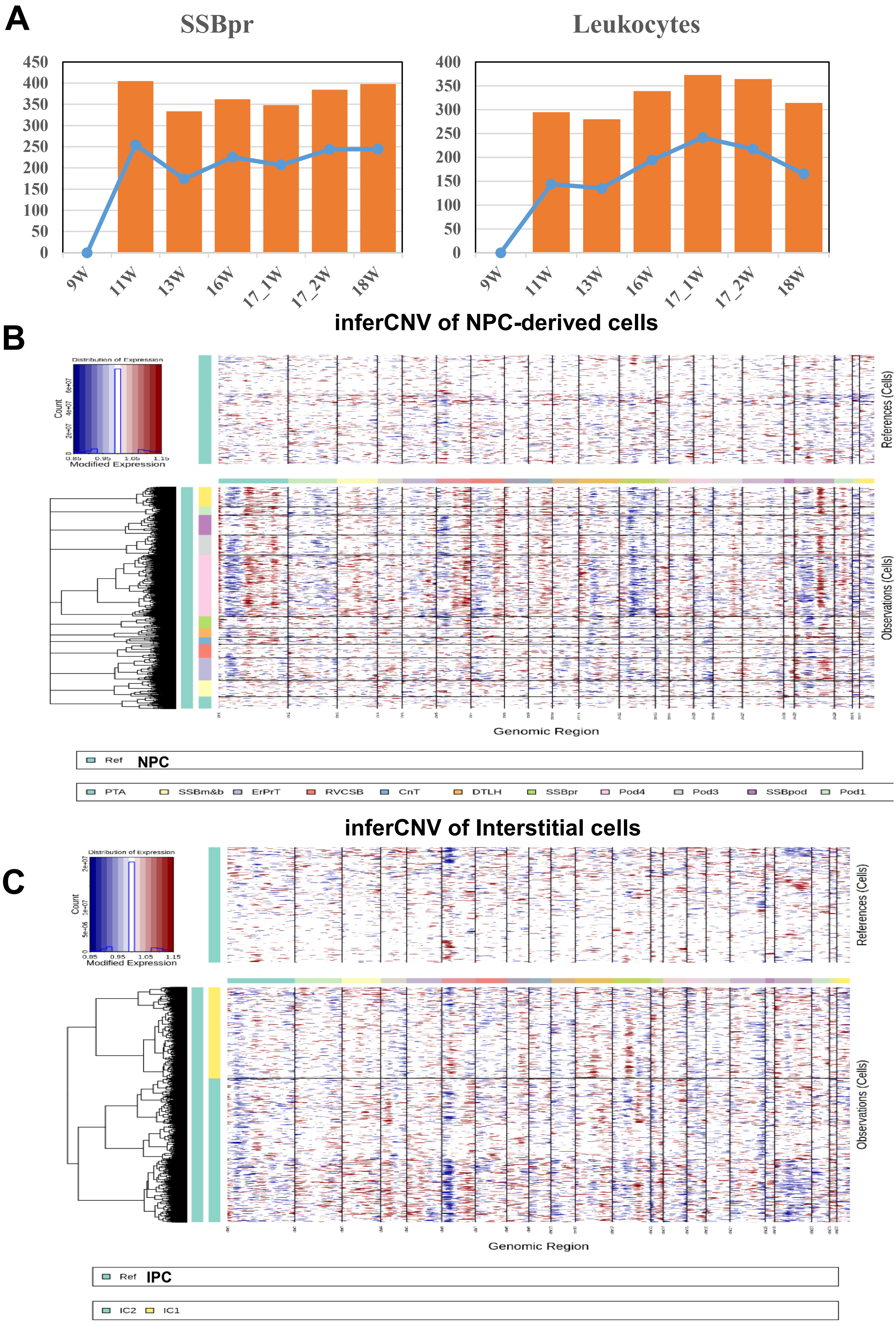

Fig.S2 (A) Expressed ATG genes per single cell in SSBpr and leukocytes. (B-C) inferCNV results of NPC and IPC derived fetal renal cells.

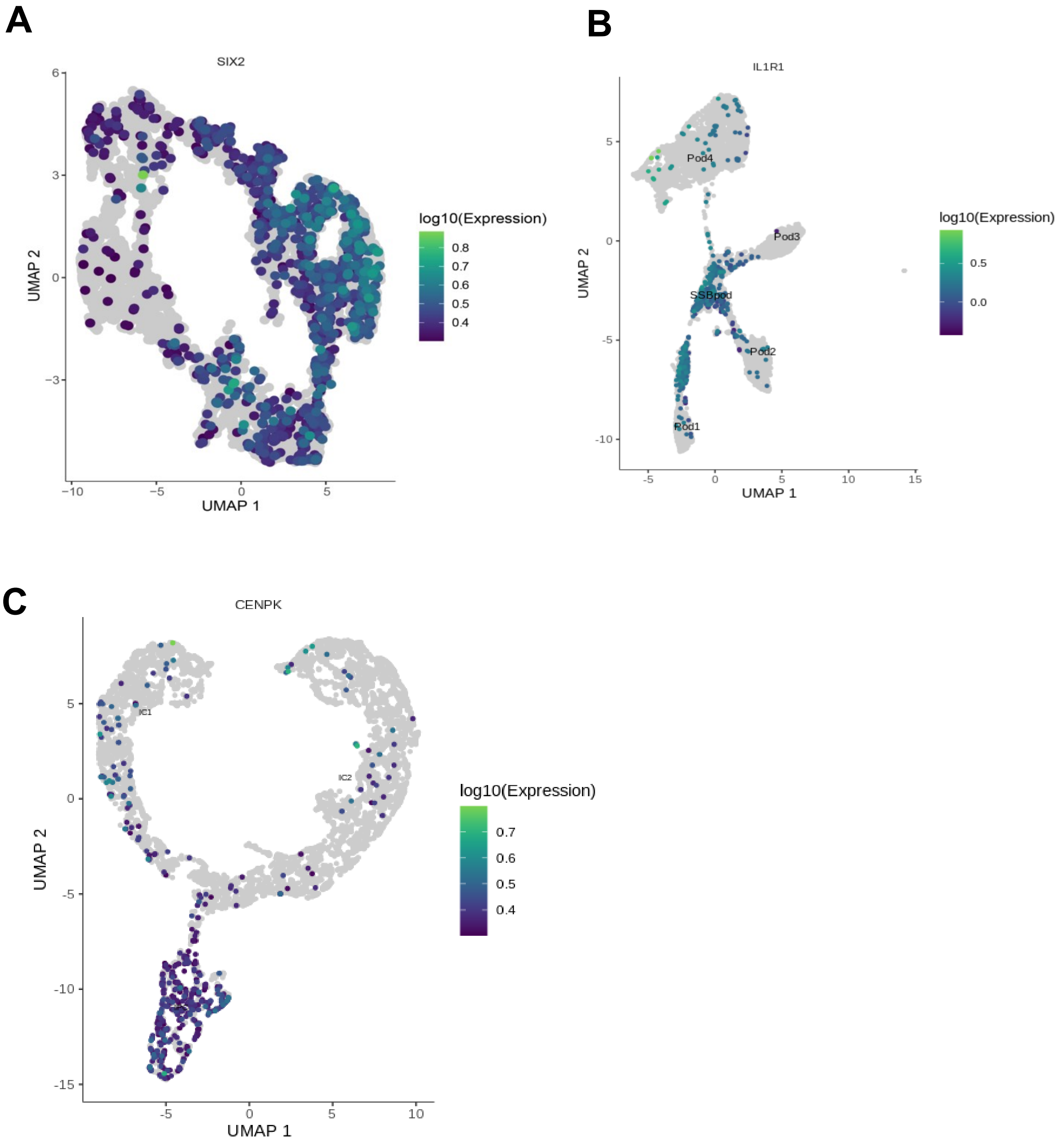

Fig.S3 (A) SIX2 expression in Monocle 3 cluster of NPCs. (B) IL1R1 expression in Monocle 3 cluster of SSBpod and pods. (IL1R1 is the marker of SSBpod). (C) CENPK expression in Monocle 3 cluster of IPC and ICs. (CENPK is the marker of IPC)

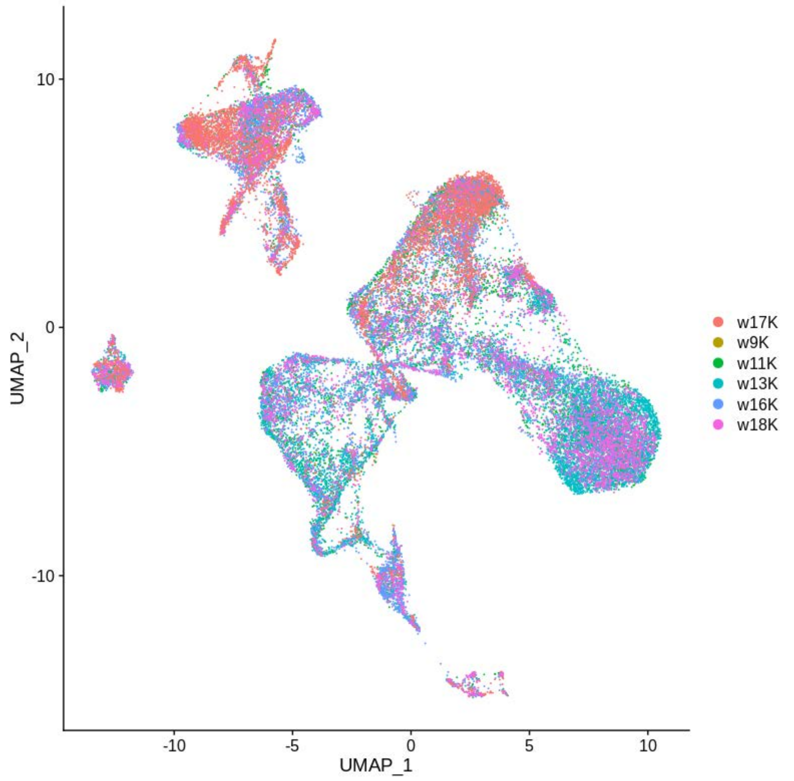

Fig.S4 UMAP plot of different samples in all weeks.
